# Which site is better for prophylactic ileostomy after laparoscopic rectal cancer surgery? By the specimen extraction site or new site: A systematic review and meta-analysis

**DOI:** 10.3389/fonc.2023.1116502

**Published:** 2023-02-15

**Authors:** Bobo Zheng, Quan Wang, Mingtian Wei, Yumin Yue, Xiaojun Li

**Affiliations:** ^1^ Department of General Surgery, Shaanxi Provincial People’s Hospital, Xi’an, China; ^2^ Ambulatory Surgery Center of Xijing Hospital, Fourth Military Medical University, Xi’an, China; ^3^ Department of Gastrointestinal Surgery, West China Hospital, Sichuan University, Chengdu, China

**Keywords:** prophylactic ileostomy, the specimen extraction site, new site, laparoscopic rectal cancer surgery, parastomal hernias

## Abstract

**Background:**

There is controversy about the outcomes of prophylactic ileostomy *via* the specimen extraction site (SES) after laparoscopic rectal cancer surgery (LRCS). We, therefore, performed a meta-analysis to determine the efficacy and safety of stoma through the SES versus new site (NS).

**Methods:**

All relevant studies from 1997 to 2022 were searched in the PubMed, EMBASE, Cochrane Library, CNKI, VIP databases. This meta-analysis was performed using RevMan software 5.3 for statistical analysis.

**Results:**

7 studies with 1736 patients were included. The present meta-analysis noted that prophylactic ileostomy *via* SES was associated with a higher risk of overall stoma-related complications, especially parastomal hernia (OR, 2.39, 95% CI 1.43-4.00; p=0.0008). No statistical difference was found in terms of wound infection, ileus, stoma edema, stoma prolapse, stoma necrosis, stoma infection, stoma bleeding, stoma stenosis, skin inflammation around the stoma, stoma retraction and postoperative pain score on postoperative day 1 and 3 between SES group and NS group. However, prophylactic ileostomy *via* SES was associated with lesser blood loss (MD = -0.38, 95% CI: -0.62 - -0.13; p=0.003), shorter operation time(MD = -0.43, 95% CI: -0.54 - -0.32 min; p<0.00001), shorter post-operative hospital stay (MD = -0.26, 95% CI: -0.43 - -0.08; p=0.004), shorter time to first flatus(MD = -0.23, 95% CI: -0.39 - -0.08; p=0.003) and lower postoperative pain score on postoperative day 2.

**Conclusion:**

Prophylactic ileostomy *via* SES after LRCS reduces new incision, decreases operative time, promotes postoperative recovery, and improves cosmetic outcomes, but may increase the incidence of parastomal hernias. The vast majority of parastomal hernias can be repaired by closing the ileostomy, therefore SES remain an option for temporary ileostomy after LRCS.

## Introduction

Colorectal cancer (CRC) remains the third most common cancer in the world (1). Surgery is still the main treatment option. With the development of laparoscopic technology, laparoscopic rectal cancer surgery (LRCS) has been widely carried out, and achieve better treatment results (2). Laparoscopic surgery was able to achieve similar disease-free survival and longer overall survival compared to open surgery (3). Laparoscopic surgery has replaced open surgery as the mainstream treatment for rectal cancer.

Anastomotic leak (AL) is a serious complication of rectal cancer surgery, associating with high local recurrence rates and poor survival (4). The incidence of anastomotic leakage after anterior resection remains disturbingly high, ranging from 3.5% - 17.0% (5–8). When performing laparoscopic low anterior resection, prophylactic ileostomy is considered to prevent AL in patients with low anastomosis levels, receiving neoadjuvant concurrent radiotherapy or at high risk of anastomotic leak due to vascular incompetence (9–11).

With the widespread use of Natural orifice specimen extraction surgery (NOSES) in laparoscopic colorectal cancer surgery(LCRCS) in recent years, it is gradually becoming recognized by more surgeons. Several studies have shown that NOSES can significantly accelerate postoperative recovery and achieve cosmetic results compared to conventional laparoscopic colorectal cancer surgery, while there is no statistical difference in 3-year overall survival time and disease-free survival time between NOSES and conventional LCRCS (12–15). For patients with rectal cancer requiring prophylactic ileostomy, an new incision in the abdomen is unavoidable, so theoretically it would be more in line with the minimally invasive concept to perform prophylactic ileostomy through specimen extraction site (SES).

Conventional LRCS requires an abdominal incision (approximately 4-8 cm) to remove the specimen, which increases the incision-related complications, including incision site infection, incisional steatosis and incisional hernia (14, 16, 17). However, there is controversy about the outcomes of prophylactic ileostomy *via* SES. Yoo SB reported that no statistical differences were found for prophylactic ileostomy through SES or new site (NS) after LRCS (18), however, several studies in recent years reported stoma through the SES was be superior than NS (19–21). We, therefore, performed a meta-analysis to determine the efficacy and safety of stoma through the SES versus NS.

## Materials and methods

The present meta-analysis follows Preferred Reporting Items for Systematic reviews and Meta-Analyses (PRISMA) reporting guidelines (22).

### Search strategy

All relevant studies from 1997 to 2022 were searched in the PubMed, EMBASE, Cochrane Library, CNKI (China National Knowledge Infrastructure Whole Article Database), VIP (http://vip.hbsti.ac.cn/) databases. The following search terms were used:

“Laparoscopic”, “rectal cancer surgery”, “low anterior resection”, “ileostomy”, “stoma”, “the specimen extraction site” and “new site”. The latest search date for this study was October 22, 2022. The search strategy was not restricted to languages.

The inclusion criteria were as follows (1): Case-control studies, including randomized controlled trials, prospective studies and retrospective studies (2), The patient underwent laparoscopic surgery for rectal cancer and had a prophylactic ileostomy (3), Study comparing the safety and efficacy of ileostomy *via* SES with NS;

The exclusion criteria were as follows (1): The studies were case reports, reviews, or comments (2), Study did not compare prophylactic ileostomy *via* SES with NS after LRCS (3). The meeting abstract did not provide detailed data.

### Outcomes of interest

The primary outcomes of interest were stoma-related complications (including parastomal hernia, stoma edema, stoma prolapse, stoma necrosis, stoma infection, stoma bleeding, stoma stenosis, skin inflammation around the stoma, stoma retraction).

The secondary outcomes were blood loss, operation time, post-operative hospital stay, time to first flatus, ileus, wound infection and postoperative pain score.

Postoperative pain score was performed by the nurse in charge using the numerical rating system (NRS).

### Data extraction and methodological quality assessment

The following data was extracted from each included study: country, year, No. of patients, age, sex, body mass index (BMI), neoadjuvant chemoradiation, pTNM, stoma site, the specimen extraction site and outcomes. The data was extracted by two authors separately, and a third author was added to discuss the decision in case of disagreement.

The methodological quality of the included literature was evaluated using the Newcastle-Ottawa Quality Assessment Scale (NOS) (23).

### Statistical analysis

We used Revman Statistical Software (Ver. 5.3 Copenhagen, Denmark) for statistical analysis. For binary data, the pooled outcomes were expressed as odds ratios (ORs) and 95% confidence intervals (95% CI). For continuous data, the pooled outcomes were reported as mean differences and 95% CI. If the article provided only the median and range, the formula described by Hozo SP was used to calculate the mean and standard deviation (24). Heterogeneity was evaluated using the i-squared statistic, and when i-squared > 50%, it was considered to have considerable heterogeneity, which was then analyzed statistically using a random-effects model, otherwise a fixed-effects model was used. A *p* value < 0.05 was considered statistically significant.

## Results

**Figure 1:**
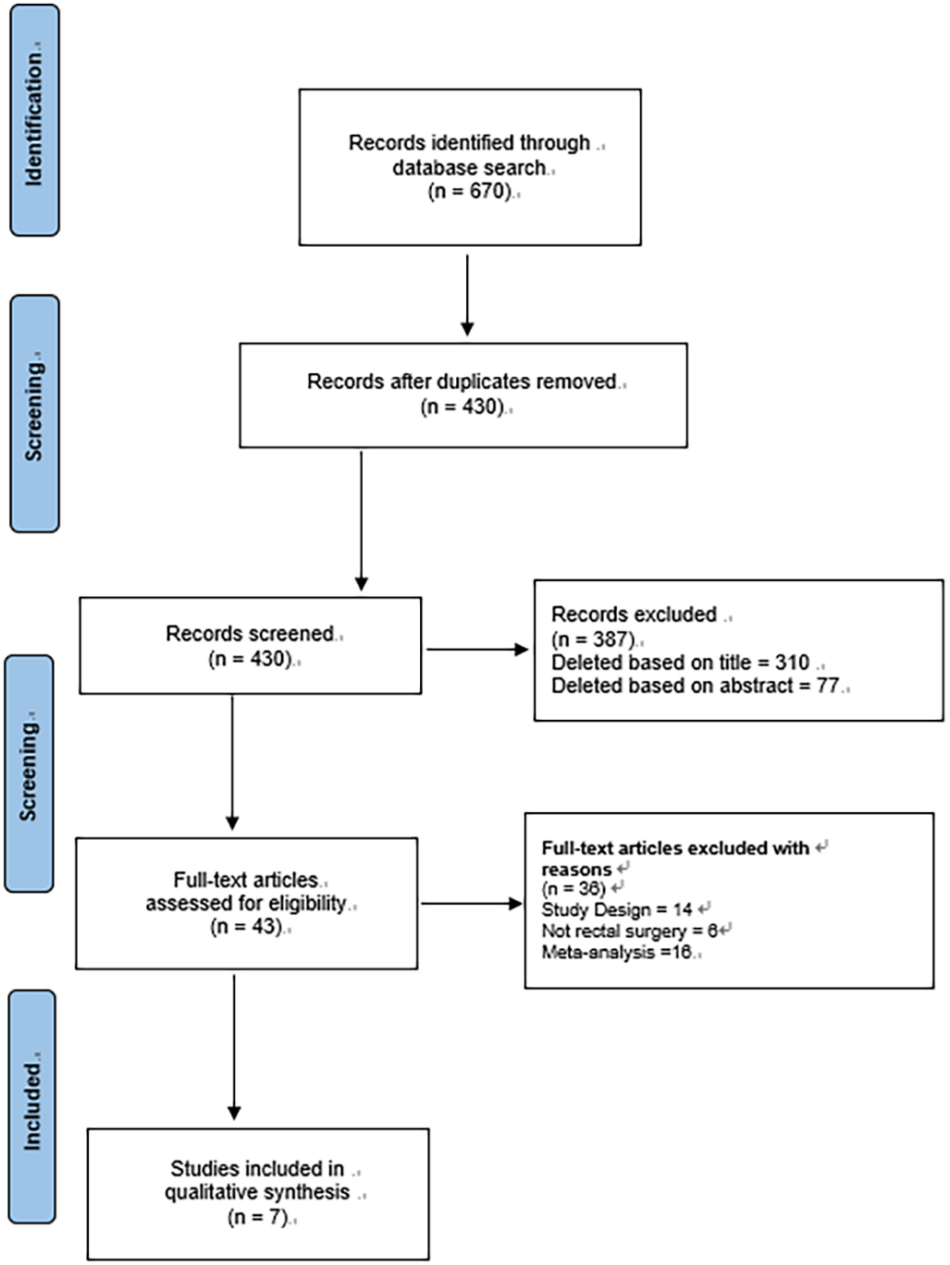
Flowchart of the screening process for included studies.

A total of 670 studies were obtained by the literature search strategy described above (**Figure 1**). After removal of duplicates, 430 literature abstracts were reviewed and assessed according to the inclusion and exclusion criteria. 43 studies were downloaded in full for screening. Seven studies were finally included for quantitative analysis. The total number of participants in the 7 included studies (18–21, 25–27) was 1736. Overall, 704 patients underwent prophylactic ileostomy *via* the SES, while 1032 patients underwent prophylactic ileostomy *via* the NS after LRCS.

### Characteristics and quality of the included studies

**Table 1** showed characteristics of the included studies. Four studies were completed in China (19, 21, 25, 27), two studies (18, 20) in Korea and one study (26) in Turkey. Three studies (21, 25, 26) performed prophylactic ileostomy at the right lower abdominal SES, two studies (18, 19) performed ileostomy at the left lower abdominal SES, one study (27) performed ileostomy at the median lower abdominal SES, and one study (20) performed ileostomy at either the left lower abdominal or right lower abdominal SES. All included studies were retrospective in design (**Table 2**). One study (27) had an NOS score of 7 and the remaining 6 studies had an NOS score of 8.

**Table 1:** Characteristics of the included studies.

		Peng D	Lee KY	Wang P	Li W	Karakayali FY	Yoo SB	Ahao W
Country		China	Korea	China	China	Turkey	Korea	China
Year		2022	2019	2018	2017	2015	2013	2022
No. of patients	SES	162	141	155	139	21	56	30
	NS	95	57	176	599	25	49	31
Age	SES	62.0 ± 10.3	62.4 ±11.4	55.5 ± 12.2	40.7 ± 13.4	61 ± 13	59.8 ± 10.5	56.4 ± 12.2
	NS	60.3 ± 11.5	63.7 ±10.9	57.0 ± 11.6	42.8 ± 15.9	67 ± 13	57.4 ± 12.2	56.7 ± 9.7
Sex(M/F)	SES	101/61	93/48	88/67	79/60	14/7	35/21	14/16
	NS	62/33	37/40	98/78	253/346	10/15	29/20	15/16
BMI (mean±SD), kg/m^2^	SES	22.9 ± 2.9	23.7 ± 0.8	23.9 ± 2.5	25.5 ± 5.6	24 ± 6	–	21.8 ± 3.2
	NS	22.9 ± 3.1	23.9 ± 1.0	24.4 ± 3.1	25.9 ± 5.9	25 ± 6	–	22.4 ± 3.2
Neoadjuvant chemoradiation	SES	48	66	48	4	9	45	1/29
	NS	25	29	52	16	7	37	4/27
pTNM(0/I/II/III/IV)	SES	0/55/54/50/3	17/46/38/32/8	0/38/61/44/12	0/2/76/59/2	0/9/5/5/2	11/18/13/13/1	0/7/10/13/0
	NS	0/39/20/31/5	5/15/15/19/3	0/45/70/52/9	0/16/371/207/5	0/12/5/6/2	8/12/9/20/0	0/8/9/14/0
Stoma site	SES	LLQ:162	LLQ:134/RLQ:7/RUQ:0	RLQ:155	RLQ:139	RLQ: 21	LLQ:56	M:30
	NS	RLQ:95	LLQ:24/ RLQ:23/ RUQ:1	RLQ:176	RLQ:599	RLQ: 25	RLR:49	RLQ:31
The specimen extraction site:	SES	LLQ:162	P:3/LLQ:134/RLQ:4/A:0	RLQ:155	RLQ:139	RLQ: 21	LLQ:56	M:30
	NS	LLQ:95	P:48/LLQ:0/ RLQ:0/A:9	LLQ:176	M:127/Pf:429/P:35/LLQ:8	Pf: 25	LLQ:49	M:31
Follow up time		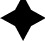	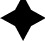	SES: 40 mo; NS:41 mo	SES: 23.2 mo; NS:32.7 mo	SES: 600 ±210 days; NS: 548 ±242 days	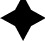	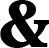
The closure time of ileotomy		NG	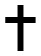 , 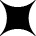 :3m	NG	SES: 4.7 m; NS:5.4 m (median)	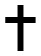 , 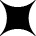 : 6-12 weeks	168 days (mean)	SES: 110 ±32 days; NS: 159.4 ±73.4 days
The kind of evaluation of parastomal hernia		NG	CT	NG	clinical documentation	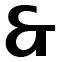	NG	NG

M/F, Male/Female; T2DM, type 2 diabetes mellitus; CHD, coronary heart disease; BMI, body mass index; SES, Specimen extraction site; NS, new site; TNM, tumor nodes metastasis; LLQ, left lower quadrant; RLQ, right lower quadrant; RUQ, right upper quadrant, P, Periumbilical; A, Anus; M, midline; Pf, Pfannenstiel; mo, Month; NG: not given; 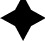: The study was followed up to the time of ileostomy return; 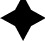: 3 months after ileostomy return; 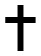: Patients who require post-operative chemotherapy will have an ileostomy after all chemotherapy has been completed; 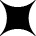 patients who do not require post-operative chemotherapy will have an ileostomy. CT, computed tomography; 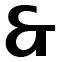: The diagnosis of a parastomal hernia was made by physical examination, radiographic imaging for various reasons and findings during ileostomy removal.

**Table 2:** Methodological quality of the included studies.

	Selection				Comparability	Exposure			Total stars
	1	2	3	4	5	6	7	8	
Peng D 2022	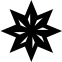	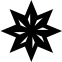	–	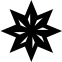	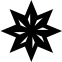 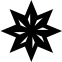	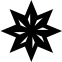	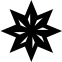	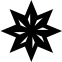	8
Ahao W 2022	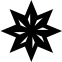	–	–	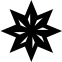	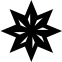 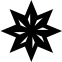	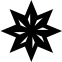	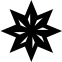	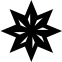	7
Lee KY 2019	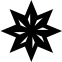	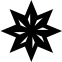	–	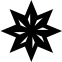	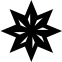 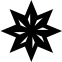	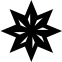	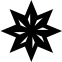	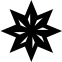	8
Wang P 2018	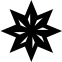	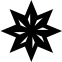	–	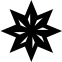	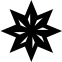 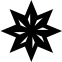	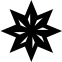	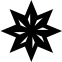	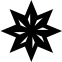	8
Li W 2017	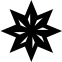	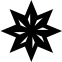	–	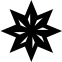	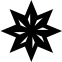 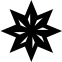	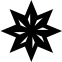	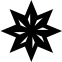	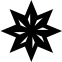	8
Karakayali FY 2015	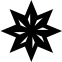	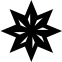	–	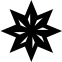	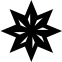 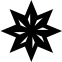	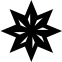	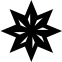	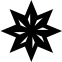	8
Yoo SB 2013	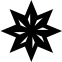	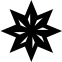	–	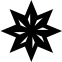	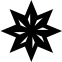 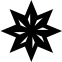	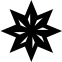	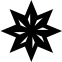	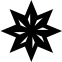	8

Is the case definition adequate?, 2 Representativeness of the cases, 3 Selection of Controls, 4 Definition of Controls, 5 Comparability of cases and controls on the basis of the design or analysis, 6 Ascertainment of exposure, 7 Same method of ascertainment for cases and controls, 8 Non-Response rate.

### Stoma-related complications

**Figure 2:**
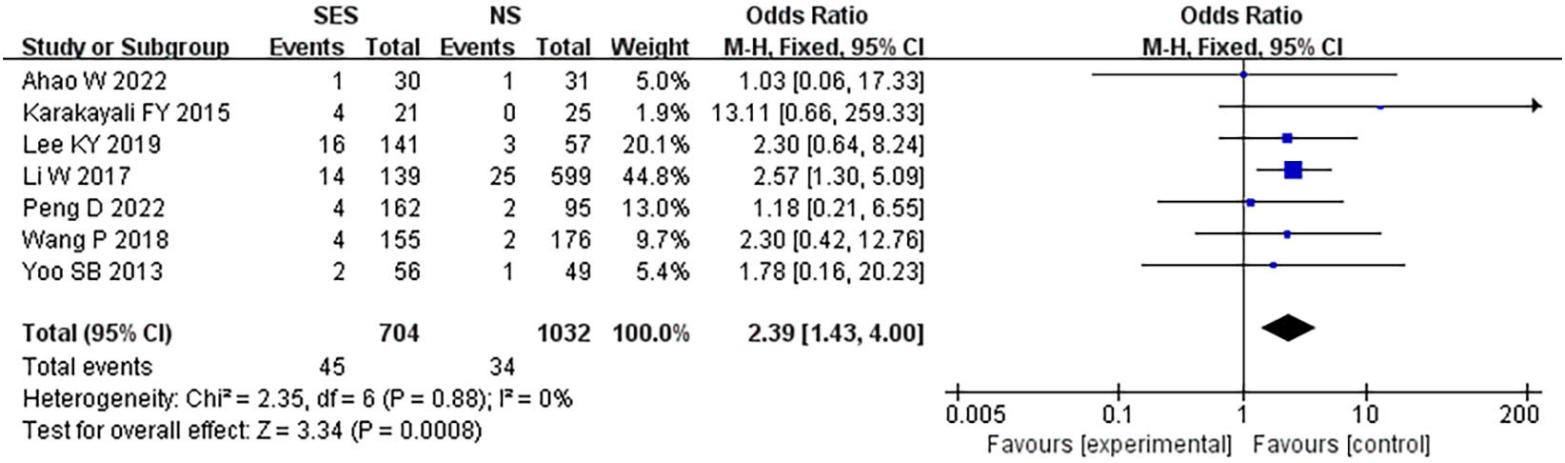
Parastomal hernia. NS: new site.

All seven studies reported parastomal hernia rates; 6.4% (45/704) in the SES group compared to 3.3% (34/1032) in the NS group. The pooled risk of parastomal hernia was higher in those with prophylactic ileostomy *via* the SES compared with NS (OR, 2.39, 95% CI 1.43-4.00; p=0.0008). No heterogeneity was found (I^2 =^ 0%) (**Figure 2**).

**Table 3** presents the results of the pooled analysis of overall stoma-related complications. The SES group was significantly higher than the NS group in terms of total stoma-related complications (OR, 2.11, 95% CI 1.37-3.26; p=0.0007; I^2 =^ 18%). There was no statistical difference between the SES and NS groups in terms of stoma edema (OR, 0.8, 95% CI 0.4-1.59; p=0.52; I^2 =^ 43%), stoma prolapse(OR, 1.93, 95% CI 0.71-5.23; p=0.20; I^2 =^ 0%), stoma necrosis(OR, 0.17, 95% CI 0.02-1.54; p=0.12; I^2 =^ 0%), stoma infection(OR, 3.39, 95% CI 0.83-13.78; p=0.09; I^2 =^ 0%), stoma bleeding(OR, 1.16, 95% CI 0.26-5.23; p=0.85; I^2 =^ 21%), stoma stenosis(OR, 1.69, 95% CI 0.45-6.35; p=0.44; I^2 =^ 45%), skin inflammation around the stoma(OR, 1.28, 95% CI 0.76-2.15; p=0.35; I^2 =^ 0%) and stoma retraction(OR, 4.09, 95% CI 0.83-20.08; p=0.08; I^2 =^ 0%).

**Table 3:** The results of the pooled analysis of stoma-related complications.

	Number of included studies	Number of patients		I^2^	OR	95% CI	P value
		SES	new site				
Stoma-related complications	3	442	751	18	2.11	1.37, 3.26	0.0007
Stoma edema	2	317	271	43	0.80	0.40, 1.59	0.52
Stoma prolapse	4	597	927	0	1.93	0.71, 5.23	0.2
Stoma necrosis	2	317	271	0	0.17	0.02, 1.54	0.12
Stoma infection	2	294	775	0	3.39	0.83, 13.78	0.09
Stoma bleeding	3	458	328	21	1.16	0.26, 5.23	0.85
Stoma stenosis	3	456	870	45	1.69	0.45, 6.35	0.44
skin inflammation around the stoma	4	488	359	0	1.28	0.76, 2.15	0.35
Parastomal hernia	7	704	1032	0	2.39	1.43, 4.00	0.0008
Stoma retraction	4	465	863	0	4.09	0.83, 20.08	0.08

### Operation time

**Figure 3:**
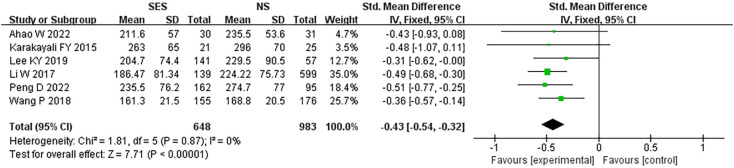
Operation time. NS: new site.

Six studies (19–21, 25–27) reported operation time. The time of operation in the prophylactic ileostomy *via* the SES was shorter than that in the prophylactic ileostomy *via* NS after LRCS (MD = -0.43, 95% CI: -0.54 - -0.32 min; p<0.00001). No heterogeneity was found (I^2 =^ 0%) (**Figure 3**).

### Blood loss

**Figure 4:**
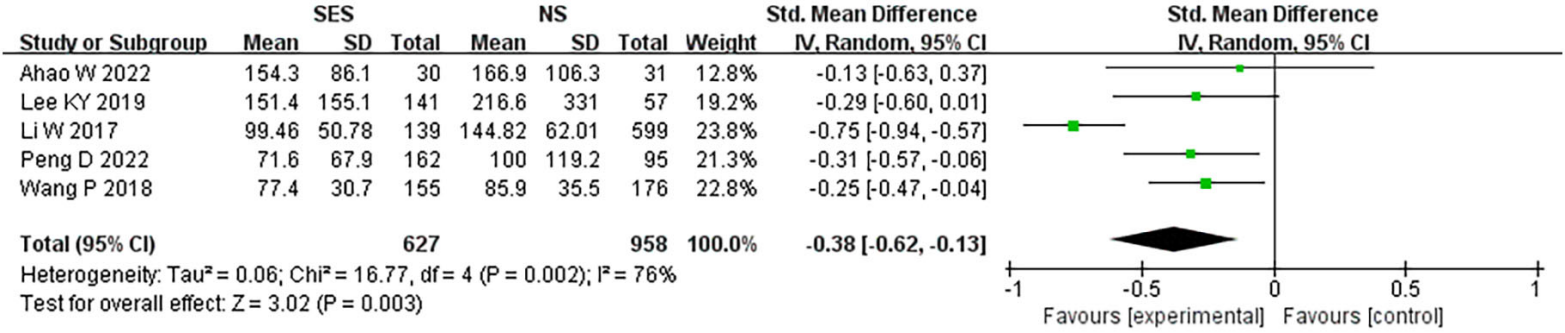
Blood loss. NS: new site.

Five studies (19–21, 25, 27) reported blood loss. A pooled analysis performed using the fixed-effects model revealed a significantly reduced blood loss in the SES group compared with the NS group (MD = -0.38, 95% CI: -0.62 - -0.13; p=0.003). High heterogeneity was detected (I^2 =^ 76%) (**Figure 4**).

### Post-operative hospital stay

**Figure 5:**
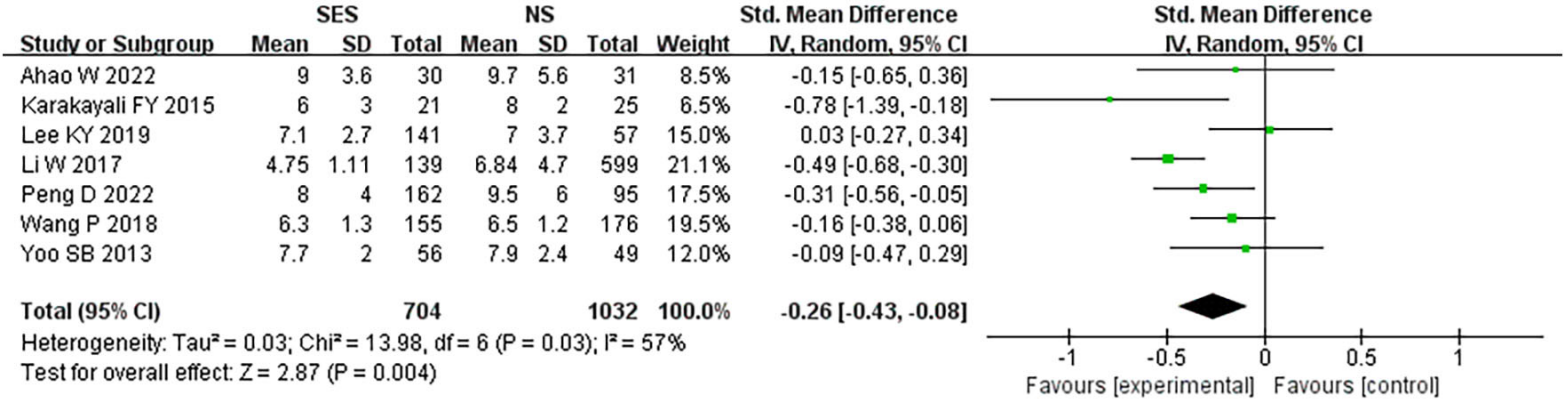
Postoperative hospital stay. NS: new site.

All seven studies reported post-operative hospital stay. This pooled analysis using a random effects model showed that the SES group had a significantly lower postoperative hospital stay than the NS group (MD = -0.26, 95% CI: -0.43 - -0.08; p=0.004). High heterogeneity was detected (I^2 =^ 57%) (**Figure 5**).

### Time to first flatus

**Figure 6:**
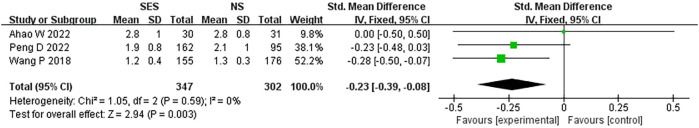
Time to first flatus. NS: new site.

Three studies (19, 21, 27) reported time to first flatus. This pooled analysis using a fixed effects model showed that the SES group had a significantly shorter time to first flatus than the NS group (MD = -0.23, 95% CI: -0.39 - -0.08; p=0.003). No heterogeneity was found (I^2 =^ 0%) (**Figure 6**).

### Ileus

**Figure 7:**
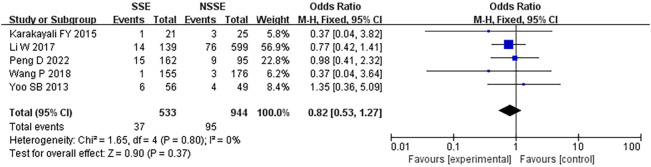
Ileus.

Five studies (18, 19, 21, 25, 26) reported ileus rate. This pooled analysis using a fixed effects model showed that there was no significantly difference between the SES group and the NS group. (OR, 0.82, 95% CI 0.53-1.27; p=0.37). No heterogeneity was found (I^2^ = 0%) (**Figure 7**).

### Wound infection

**Figure 8:**
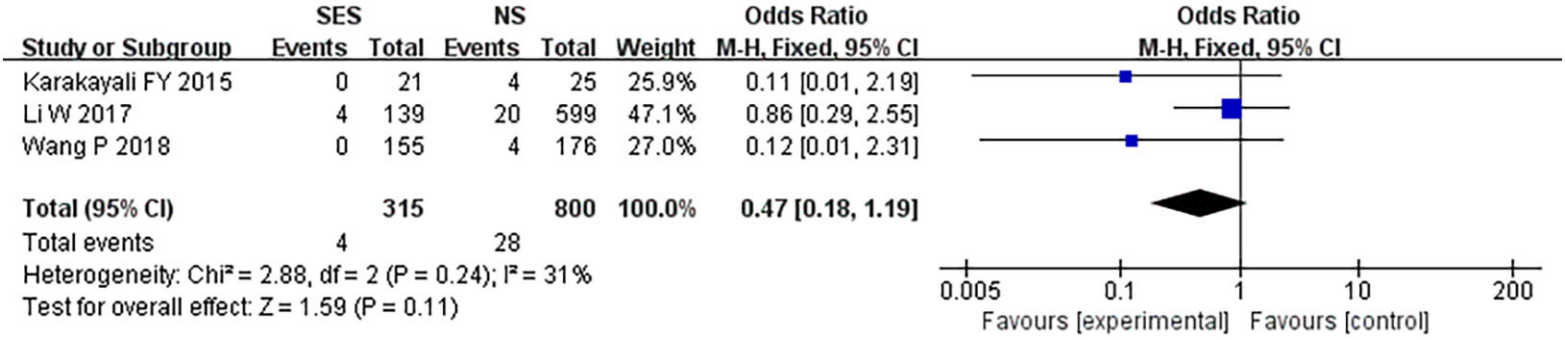
Wound infection. NS: new site.

Three studies (21, 25, 26) reported wound infection; 1.4% (4/315) in the SES group compared to 3.5% in the NS group. A pooled analysis using the fixed-effects model revealed no significant difference in wound infection rates between the two groups (OR, 0.47, 95% CI 0.18–1.19; p=0.11). Moderate heterogeneity was found (I^2 =^ 31%) (**Figure 8**).

### Postoperative pain score

**Figure 9:**
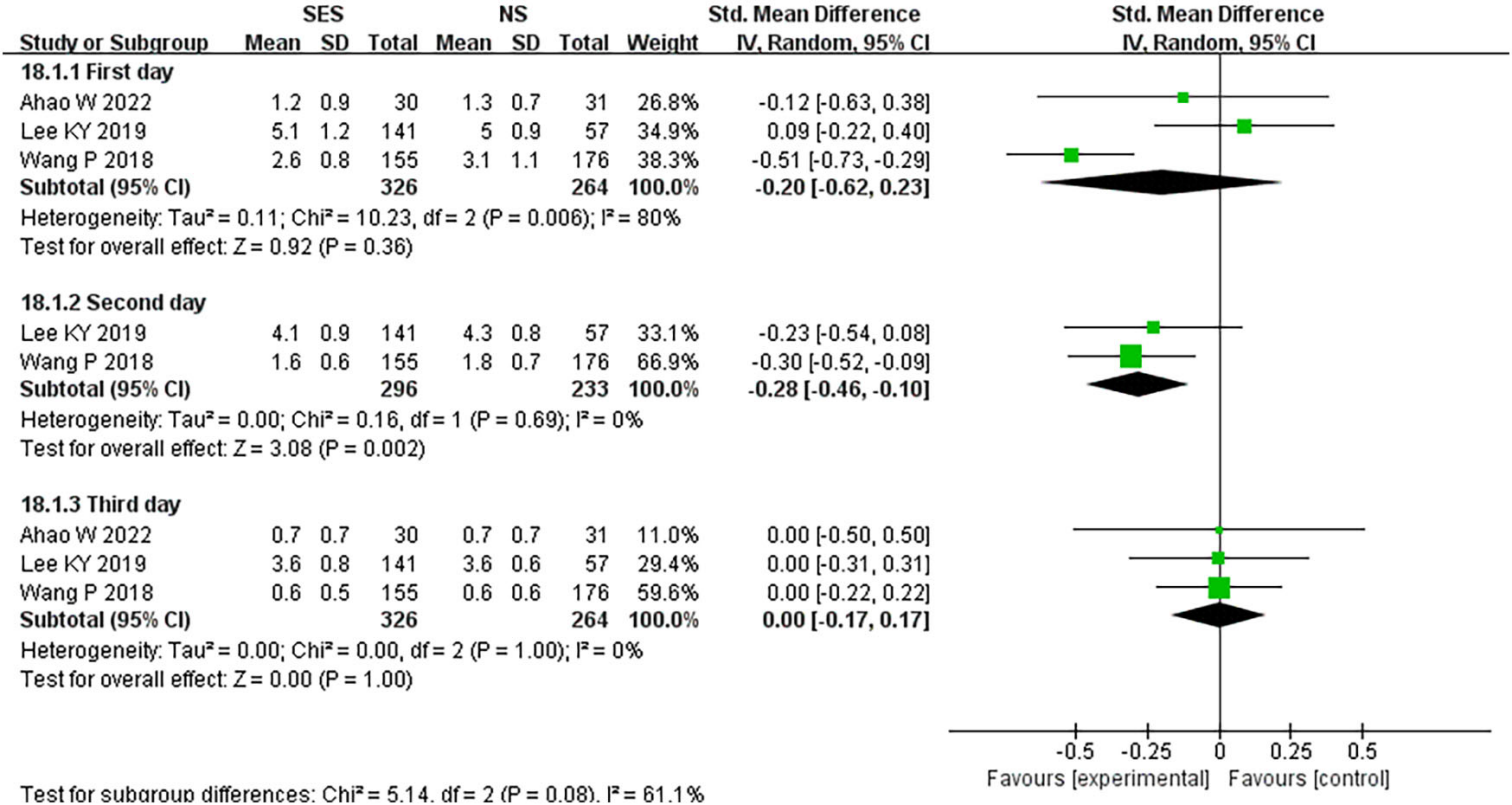
Postoperative pain. NS: new site.

Three studies (20, 21, 27) reported postoperative pain score. This pooled analysis using a random effects model showed that there was no statistical difference in postoperative pain score between the SES and NS group on postoperative day 1(MD = -0.20, 95% CI: -0.62 -0.23; p=0.36) and postoperative day 3(MD = 0.00, 95% CI: -0.17 – 0.17; p=1.00), while on postoperative day 2, postoperative pain score was significantly lower in the SES group than in the NS group(MD = -0.28, 95% CI: -0.46 – 0.10; p=0.002). High heterogeneity was detected on postoperative day 1 (I^2 =^ 80%) (**Figure 9**).

## Discussion

To our knowledge, this was the first meta-analysis comparing prophylactic ileostomy through SES with NS after LRCS. The present meta-analysis noted that prophylactic ileostomy *via* SES was associated with a higher risk of overall stoma-related complications, especially parastomal hernia. We observed no statistical difference in terms of wound infection, ileus, stoma edema, stoma prolapse, stoma necrosis, stoma infection, stoma bleeding, stoma stenosis, skin inflammation around the stoma, stoma retraction and postoperative pain score on postoperative day 1 and 3 between SES group and NS group. However, prophylactic ileostomy *via* SES was associated with lesser blood loss, shorter operation time, shorter post-operative hospital stay, shorter time to first flatus and lower postoperative pain score on postoperative day 2.

Anastomotic leak (AL) is the most dreaded postoperative complication of colorectal cancer, with an incidence ranging from 1% to 21%, and it has a serious negative impact on the patient’s postoperative recovery, quality of life and survival (28–31). There are many risk factors for AL, such as longer operation time, use of more than 3 staples, ultra-low anastomosis, male, neoadjuvant radiotherapy (32–35). Prophylactic ileostomy has been proven and accepted by colorectal surgeons to prevent postoperative AL in rectal cancer and to reduce the adverse consequences of anastomosis (36–38). Conventional laparoscopic rectal cancer surgery is followed by prophylactic ileostomy through a new incision other than the one from which the specimen was taken. Theoretically, if prophylactic ileostomy is performed through the specimen retrieval incision, it will reduce the number of abdominal wall incisions and thus be more in line with the minimally invasive concept.

There is still no consensus on the definition of a parastomal hernia, which has caused the incidence of parastomal hernia to fluctuate from 1% to 50% as reported in the literature (39). One study reported that parastomal hernias were detected by clinical examination, imaging or during ileostomy reversal (26), while recent literature has reported that parastomal hernias were diagnosed by CT scan (20). The present meta-analysis reported a 6.4% incidence of parastomal hernia in the SES group compared to a 3.3% incidence of parastomal hernia in the NS group. Although the incidence of parastomal hernias was significantly higher in the SES group than in the NS group, all parastomal hernias could be resolved by ileostomy reversal surgery, and parastomal hernias had a minor impact on clinical prognosis (25).

The present study reported a significantly shorter operative time in the SES group compared with the NS group; This may be due to the fact that a temporary ileostomy through the specimen retrieval site reduces the number of surgical steps required to close the new incision; and results in a shorter operative time.

Our study also has limitations: first, all the studies included in the current meta-analysis were retrospective studies, which may have some influence on the results to some extent. Second, only short-term outcomes were included in this study, and long-term indicators such as survival were not analyzed; this is because the original studies reported very few long-term survival data. Third, due to the inconsistency in the definition of parastomal hernia, this may have had some impact on the results. Fourth, there was some variation in the location of the stoma and the method of stoma between studies, thus potentially affecting outcomes. However, the greatest strength of this meta-analysis is that it is the first study to examine the effectiveness and safety of prophylactic ileostomy after laparoscopic rectal cancer through SES.

## Conclusion

Prophylactic ileostomy *via* SES after LRCS reduces new incision, decreases operative time, promotes postoperative recovery, and improves cosmetic outcomes, but may increase the incidence of parastomal hernias. The vast majority of parastomal hernias can be repaired by closing the ileostomy, therefore SES remain an option for temporary ileostomy after LRCS.

## Data availability statement

The original contributions presented in the study are included in the article/supplementary material. Further inquiries can be directed to the corresponding author.

## Author contributions

BZ and XL contributed to the conception and design of the work. BZ, QW and MW conducted the literature search and extracted the data. YY was involved in the resolution of all the arguments. BZ and QW conducted the data analysis and wrote the manuscript. BZ and XL performed critical revision for this manuscript. All authors contributed to the article and approved the submitted version.
